# Selenium and iron, two elemental rivals in the ferroptotic death process

**DOI:** 10.18632/oncotarget.25295

**Published:** 2018-04-27

**Authors:** Irina Ingold, Marcus Conrad

**Affiliations:** Helmholtz Zentrum München, Institute of Developmental Genetics, Neuherberg, Germany

**Keywords:** selenium, ferroptosis, GPX4, cell death, transgenic mice, Autophagy

**In our present work, we show that the trace element selenium as part of glutathione peroxidase 4 (GPX4) has been a pivotal prerequisite in mammals to allow the development of complex nervous systems by protecting polyunsaturated fatty acid (PUFA)-enriched phospholipids from iron-dependent ferroptotic cell death** [4].

Two hundred years ago (1817), the Swedish scientist Jöns Jacob Berzelius discovered the trace element selenium, which he named after the Greek goddess of the moon Selene. First being considered as a toxic or even carcinogenic compound, this view drastically changed 60 years ago when Schwarz and Foltz identified an until then unknown/unspecified compound “Factor 3” as a selenium compound preventing liver necrosis upon vitamin E deficiency in rats. Nowadays, it is accepted that selenium is an essential trace element in mammals. After the identification of the first selenoprotein, glutathione peroxidase 1 (GPX1), it has become evident that selenium confers its vital function in biological systems in form of the 21st amino acid selenocysteine (Sec).

The human selenoproteome contains 25 proteins of which half of them act as oxidoreductases to maintain redox homeostasis in cells. In this respect, enzymes of the GPX family of proteins - five of them are indeed selenoproteins (GPX1-4 and GPX6) - are some of the most relevant ones due to their broad substrate specificity for different peroxides. Among these, and perhaps all selenoproteins, GPX4 is unique for its activity to reduce complex lipid peroxides and its function to control ferroptosis [6], a recently described form of cell death characterized by iron- dependent lipid peroxidation.

The non-canonical mechanism of Sec incorporation at the opal codon UGA, which is present in all three kingdoms of life, leaves the expression of selenoproteins a highly complex and energetically costly process. Yet, selenium-containing GPX4 can be found in all vertebrates, while invertebrates and plants usually express cysteine (Cys)-containing homologs. Hence, this raised the question what the actual advantage of selenolate- vs. thiolate-based catalysis actually is.

Knockout studies targeting individual selenoproteins in mice provided evidence that the conditional loss of *Gpx4* often phenocopied the simultaneous knockout of all selenoproteins in the same tissues (induced by targeting *Trsp* [*n-TUtca2*, *nuclear encoded tRNA selenocysteine 2 (anticodon TCA)*] encoding the Sec-specific tRNA), again highlighting the outstanding function of GPX4 for cell survival and tissue protection. Actually, years before the term ferroptosis was introduced, it was demonstrated that the inducible deletion of *Gpx4* triggers a caspase-independent form of cell death [5], which culminated in the establishment of GPX4 as the key regulator of this death pathway [2, 7].

To illuminate the still enigmatic question of the biological relevance of selenolate- vs. thiolate- based catalysis *in vivo*, we focused on GPX4 and generated mice with a replacement of the active site Sec to Cys. Previous work from our laboratory already showed that a redox-inactive Sec to serine substitution in GPX4 does not rescue early embryonic lethality as reported for *Gpx4-/-* mice [3]. By stark contrast, animals only expressing the GPX4- Cys variant (on a mixed genetic background) developed normally and were born. Yet, they failed to survive beyond the 3rd week after birth due to severe epileptic seizures [4]. Parvalbumin-positive interneurons, a specific subtype of GABAergic inhibitory neurons, were identified as the cells requiring selenium in GPX4, thereby presenting the limiting factor for mammalian life. Interestingly, GPX4- Cys animals on a congenic *C57BL/6J* background died during mid-gestation, albeit still substantially later than *Gpx4-/-* animals, suggesting modifier genes on the different genetic backgrounds.

**Figure 1:**
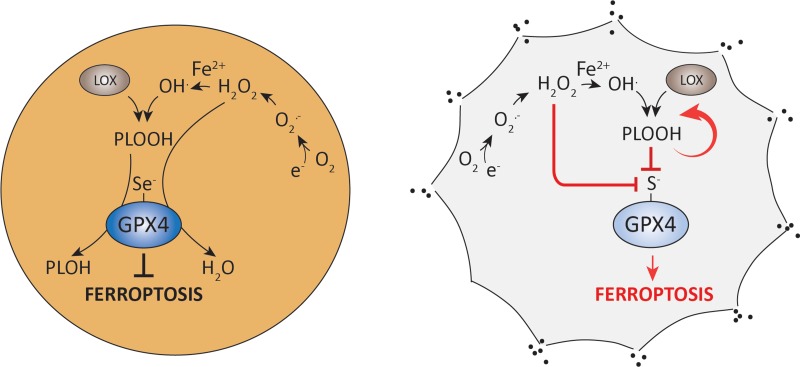
Selenium utilization by GPX4 prevents from peroxide-induced enzyme overoxidation and ferroptotic cell death Selenium-containing wildtype GPX4 efficiently reduces peroxides to their corresponding alcohols, thereby preventing from ferroptotic cell death (left). Cells expressing a selenocysteine to cysteine variant of GPX4 are highly sensitive to low peroxide concentrations due to irreversible overoxidation of the catalytically active site thiolate (right) (Abbreviations: PLOOH, phospholipid hydroperoxide; LOX, lipoxygenase; Se, selenium, S, sulfur; O_2_^.-^, superoxide anion, H_2_O_2_, hydrogen peroxide).

To elucidate the molecular underpinnings of the selenium requirement by GPX4, mouse embryonic fibroblasts were established [4]. Findings obtained by biochemical and cellular studies unveiled that sulfur-containing GPX4 is readily and irreversibly overoxidized by low peroxide concentrations and mitochondrial complex I inhibitors *in vivo*, thereby causing ferroptotic cell death (Figure 1). Notably, another important ferroptosis player (i.e. ACSL4, acyl-CoA synthetase long-chain family member 4) was found to be strongly downregulated in GPX4-Cys cells. This suggests compensatory mechanisms to reduce the cellular content of PUFAs in membranes, therefore lowering the risk of lethal lipid peroxidation as reported for *Acsl4-/-* cells [1]. We could further show that CRISPR/Cas9-mediated knockout of the *Trsp* gene in GPX4-Cys expressing cells was feasible, implying that selenoproteins are not required for cell survival and proliferation as long as a residual GPX4 activity is retained. Hence, it is tempting to speculate that selenium-containing GPX4 was an evolutionary requirement in mammals (vertebrates) to allow the utilization of PUFA-enriched membranes for the development of complex neuronal circuits, for increased cellular plasticity and for the utilization of peroxides as second messengers in redox signaling processes. Yet, an increased PUFA content in membranes comes with a burden, the increased risk towards lipid peroxidation and ferroptosis.
